# Development and evaluation of a comprehensive QA phantom for image quality and geometrical accuracy in CT and CBCT for radiotherapy

**DOI:** 10.1002/acm2.70239

**Published:** 2025-08-21

**Authors:** Minsoo Chun, Jaewon Hong, Jin Hwa Choi, Chang‐Heon Choi, Jung‐in Kim, Hong‐Gyun Wu, Minjae Choi, Na Young Jang, So‐Yeon Park

**Affiliations:** ^1^ Department of Radiation Oncology Chung‐Ang University Gwang Myeong Hospital Gyeonggi‐do Republic of Korea; ^2^ Institute of Radiation Medicine Seoul National University Medical Research Center Seoul Republic of Korea; ^3^ Department of Radiation Oncology Chung‐Ang University College of Medicine Seoul Republic of Korea; ^4^ Paprica Lab Co., Ltd Seoul Republic of Korea; ^5^ Department of Radiation Oncology Seoul National University Hospital Seoul Republic of Korea; ^6^ Department of Radiation Oncology Seoul National University College of Medicine Seoul Republic of Korea; ^7^ Project Group of the Gijang Heavy Ion Medical Accelerator Seoul National University Hospital Seoul Republic of Korea; ^8^ Department of Radiation Oncology Veterans Health Service Medical Center Seoul Republic of Korea

**Keywords:** geometrical accuracy, image quality, image‐guided radiation therapy, quality assurance phantom

## Abstract

**Purpose:**

This study presents MZ CUBE, a newly developed multipurpose phantom designed to assess both image quality and geometrical accuracy in image‐guided radiotherapy.

**Methods:**

The phantom is a 16 × 16 × 16 cm^3^ cube made of acrylonitrile butadiene styrene and designed to evaluate HU constancy, HU uniformity, spatial integrity, spatial resolution, low contrast resolution, and geometrical accuracy. Two CT simulators (Big Bore RT and Discovery RT Gen3) and two cone‐beam CT (CBCT) systems (XVI and OBI) were used for verification alongside commercial phantoms (ACR 464 and CatPhan 504).

**Results:**

HU constancy differences were within 3 HU, and uniformity was within 1 HU for CT and 2 HU for CBCT, based on repeated MZ CUBE measurements. Spatial resolution with the MZ CUBE was 7 lp/cm (Big Bore RT), 8 lp/cm (Discovery RT Gen3), 7 lp/cm (XVI), and 8 lp/cm (OBI). The minimum discernible disk diameters were 7, 5, 6, and 6 mm, respectively. Geometrical accuracy was confirmed with translational offsets below 1 mm and rotational deviations below 0.2° for both CBCT systems.

**Conclusions:**

MZ CUBE enables comprehensive image quality and geometric accuracy evaluation in a single scan, offering an efficient and cost‐effective solution for radiotherapy QA.

## 1 INTRODUCTION

Cone‐beam CT (CBCT), commonly used in image‐guided radiation therapy (IGRT), plays a critical role in verifying patient positioning and adapting to anatomical changes.^1^ To ensure its safe and accurate use in treatment delivery, comprehensive quality assurance (QA) of CBCT image quality and geometrical accuracy is essential.^2^, ^3^, ^4^, ^5^

Commercially available QA phantoms often assess image quality and geometrical accuracy separately, increasing the burden on clinical workflow and staff resources. To overcome these limitations, we developed a multipurpose phantom, the “MZ CUBE (Paprica Lab Co., Ltd., Seoul, Republic of Korea)”, capable of assessing both geometrical accuracy and image quality.

This study presents the design, fabrication, and validation of the MZ CUBE phantom. Its clinical applicability was evaluated using two CT simulators and two CBCTs.

## 2 MATERIALS AND METHODS

### 2.1 MZ CUBE specification

The phantom's main body was made of acrylonitrile butadiene styrene (ABS) for its lightweight and water‐equivalent properties,^6^ and was fabricated using CNC milling. It has a cube shape (16 × 16 × 16 cm^3^) and weighs 4.24 kg. It consists of four modules: HU constancy/spatial integrity, spatial resolution, low contrast, and geometrical accuracy. Table 1 compares the specifications of the MZ CUBE with those of conventional QA phantoms.

**TABLE 1:** Comparison of phantom specifications.

		MZ Cube	ACR 464	CatPhan 504	MIMI™
Manufacturer		PapricaLab	SunNuclear	The Phantom Laboratory	Standard Imaging
Shape		Cube	Cylinder	Cylinder	Cube
Dimension (cm)		16 × 16 × 16 (W × L × D)	20 (d) × 16 (L)	20 (d) × 25 (L)	14×14×14 (W×L×D)
Material		ABS	Solid Water	PMMA	Polystyrene
Weight (kg)		4.24	5.3	7.4	2.8
Image Quality Module					
HU constancy	Materials	Air, PE, Acrylic, Teflon	Air, PE, Acrylic, Bone	Air, PMP, LDPE, PE, Acrylic, Delrin, Teflon	N/A
	Diameter (cm)	2.5	2.5	1.25	
Spatial resolution	Materials	Air	6061 Aluminum and Polystyrene	Beads	N/A
	Line pair configuration (lp/cm)	1–10	4–12	1–21	
Low contrast resolution	Materials	Urethan polymer	Not provided	Not provided	N/A
	Diameter (mm)	25, 15, 12, 10, 7, 6, 5, 3	25, 6, 5, 4, 3, 2	15, 9, 8, 7, 6, 5, 4, 3, 2	
Positioning/repositioning module					
Contents		10 air ellipses	N/A	N/A	5 PVC rods

**FIGURE 1:**
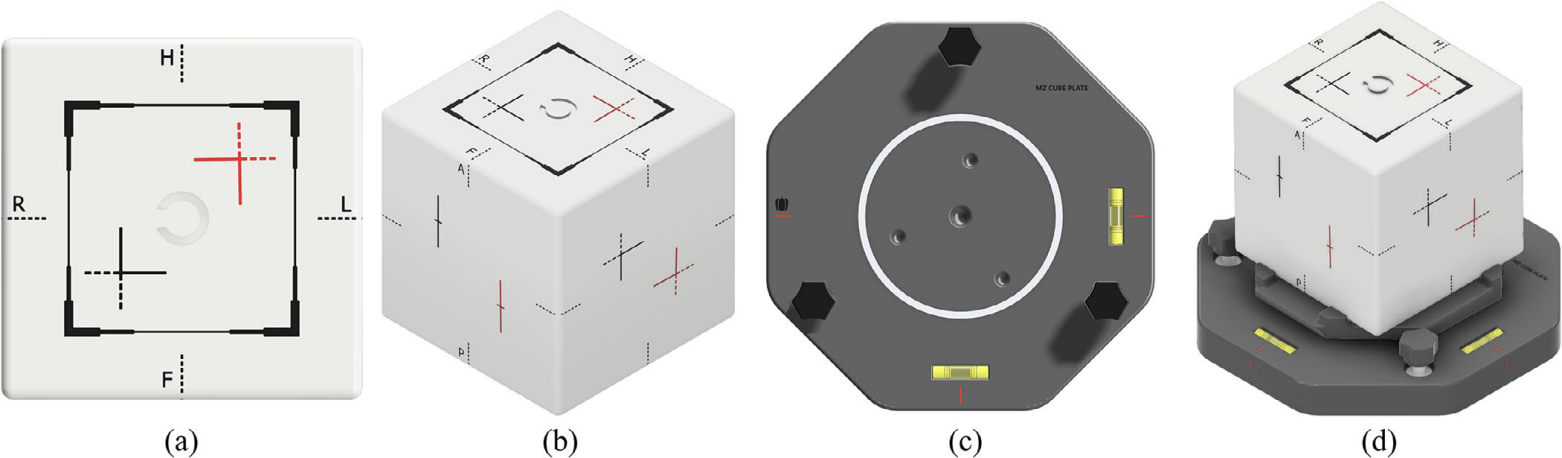
External views of MZ CUBE: (a) top, (b) side, (c) alignment plates, (d) on tilt plate.

A pre‐angled tilt plate was incorporated to introduce fixed rotational offsets. Figure 1 illustrates the phantom and its tilt plate configuration. Module‐specific design and validation details are provided in the following sections.

### 2.2 Phantom production procedure

HU‐electron density consistency is vital for treatment planning and CBCT‐based adaptive RT.^5^, ^7^, ^8^ The HU constancy module contains four 2.5‐cm‐diameter holes filled with acrylic, polyethylene, Teflon, and air, with respective electron densities of 0.004, 3.335, 3.833, and 6.243 (×10^2^ ^3^/cm^3^). The same slice includes four 2‐mm holes spaced 10 cm apart, used to assess spatial integrity.

Spatial resolution was assessed using ten air‐filled line‐pair patterns (1–10 lp/cm). Low contrast was evaluated using holes (3–25 mm diameter) filled with VytaFlex 20 (Smooth‐On, Macungie, PA), a urethane polymer with low contrast to ABS. It was mixed 1:1, degassed, poured, and cured in the module. The geometrical accuracy module contains a 2‐mm Teflon ball and ten air‐filled ellipses aligned vertically and horizontally.

Once each module was produced, they were compressed, followed by painting, engraving, and inking. The exterior includes setup‐direction indicators, field‐size markers, and offset markers. The field‐size marker confirms a 10 × 10 cm^2^ field at 100 cm SSD, with 1, 2, and 4 mm tolerance lines (Figure 1a). Two offset markers were included: black for translation‐only (2.7, 2.4, 2.0 cm in *x*, *y*, *z*), and red for combined translation and rotation (1.0° yaw, 1.3° pitch, 0.8° roll). Rotational offsets were applied using the pre‐angled tilt plate.

### 2.3 Validation

Two CT and CBCT systems were evaluated. The CT systems were (1) Big Bore RT CT (Philips), which used 250 mAs with iDose, and (2) Discovery RT Gen3 (GE), which used 350 mAs with ASiR. The CBCT systems were (1) XVI on a Versa HD (Elekta AB), paired with the Big Bore RT CT and operated at 120 kVp, 132 mAs, using an F1 bow‐tie filter and FDK reconstruction, and (2) OBI on a TrueBeam STx (Varian Medical Systems), paired with the Discovery RT Gen3 and operated at 100 kVp, 150 mAs, using a half bow‐tie filter and standard reconstruction.

#### 2.3.1 Image quality assessment

Image quality was evaluated by measuring deviations from baseline values,^2^, ^3^ which were re‐assessed after one week under identical conditions using MIM Software (v7.3.7; Cleveland, OH).

HU constancy was assessed with 2 cm^2^ ROIs, referencing ACR CT QC Manual tolerances.^9^ HU uniformity was defined as the maximum HU difference between peripheral and central ROIs in a homogeneous slice, with a 5 HU tolerance.^9^, ^10^, ^11^, ^12^ Spatial and low‐contrast resolution were determined by the visible line pairs and smallest discernible disk, as evaluated by two independent reviewers to minimize bias.^9^, ^10^, ^12^

#### 2.3.2 Geometrical accuracy assessment

**FIGURE 2:**
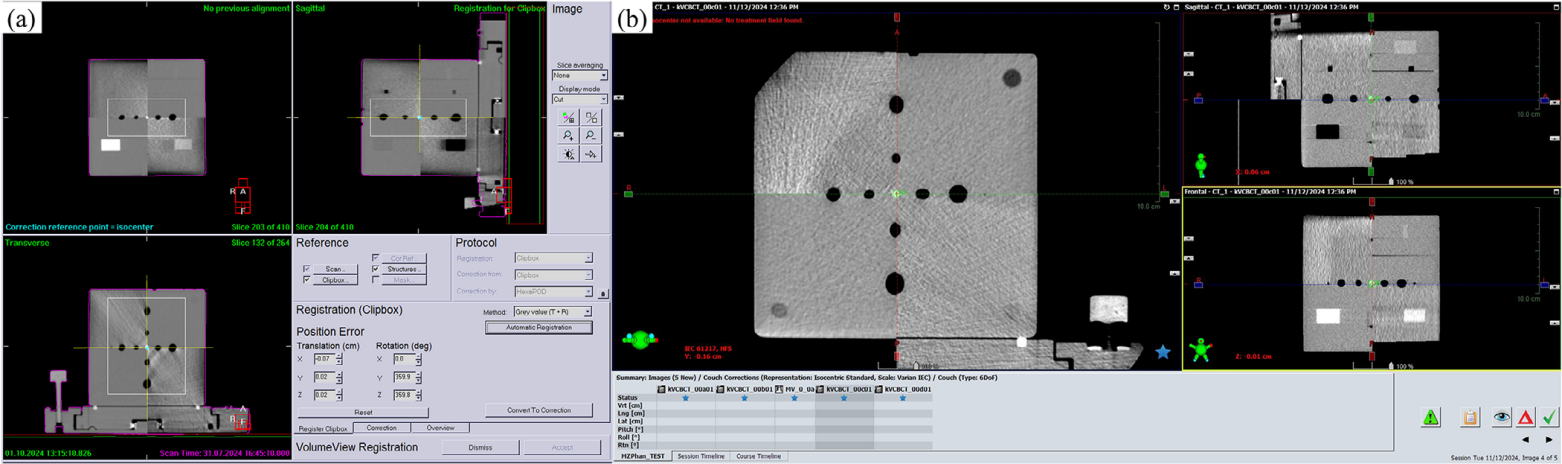
Image registration for coincidence check using (a) XVI CBCT and (b) OBI CBCT.

Spatial integrity was evaluated by measuring distances between 2‐mm holes in the HU constancy module.^3^, ^4^ After CT acquisition, the phantom origin was defined in the TPS (Monaco or Eclipse) and aligned using the offset marker. CBCT images were registered to the baseline CT (Figure 2), and differences between known and measured offsets were recorded as positioning/repositioning errors.

Coincidence between imaging and treatment coordinates was assessed by aligning the phantom to room lasers and measuring the offset between the Teflon ball center and crosshair intersection.

#### 2.3.3 Cross validation with other commercial phantoms

The ACR 464 was used for Big Bore RT CT and XVI, and CatPhan 504 for Discovery RT and OBI, using the same scan parameters as MZ CUBE. Each phantom was measured twice under identical conditions for cross‐comparison. Geometrical accuracy was cross‐validated using the MIMI Phantom (Standard Imaging, Inc.), evaluating only translational offsets due to the absence of a HexaCheck module.

#### 2.3.4 Verification of miscellaneous details

The phantom was placed at 100 cm SSD, and alignment of the 1, 2, and 4 mm tolerance markers was visually verified using a ruler with collimator settings of 10.1, 10.2, and 10.4 cm. The phantom's compatibility with four SGRT systems (AlignRT Gen5 and Horizon, ExacTrac Dynamic, and Catalyst^+^HD) was evaluated by importing CT datasets into each system and aligning the phantom to the treatment isocenter. Technical specifications are shown in Table 2.

**TABLE 2:** Key specifications of SGRT systems evaluated in this study.

	AlignRT Gen5	AlignRT Horizon	ExacTrac Dynamic	Catalyst^+^HD
**Camera Type**	Structured light + 3 stereo cameras	Structured light + 3 stereo cameras	Stereo cameras + thermal IR camera	Structured light + 3 cameras
**Field Coverage**	2048 × 1024 mm	2048 × 1024 mm	645 × 490 mm	1000 × 1800 mm
**Frame Rate**	27 fps	35 fps	18–20 fps	165 fps
**Wavelength**	660 nm	740 nm	Optical: 459 nm Thermal: 7.5–13 µm	528–624 nm

## 3 RESULTS

### 3.1 Image quality

**FIGURE 3:**
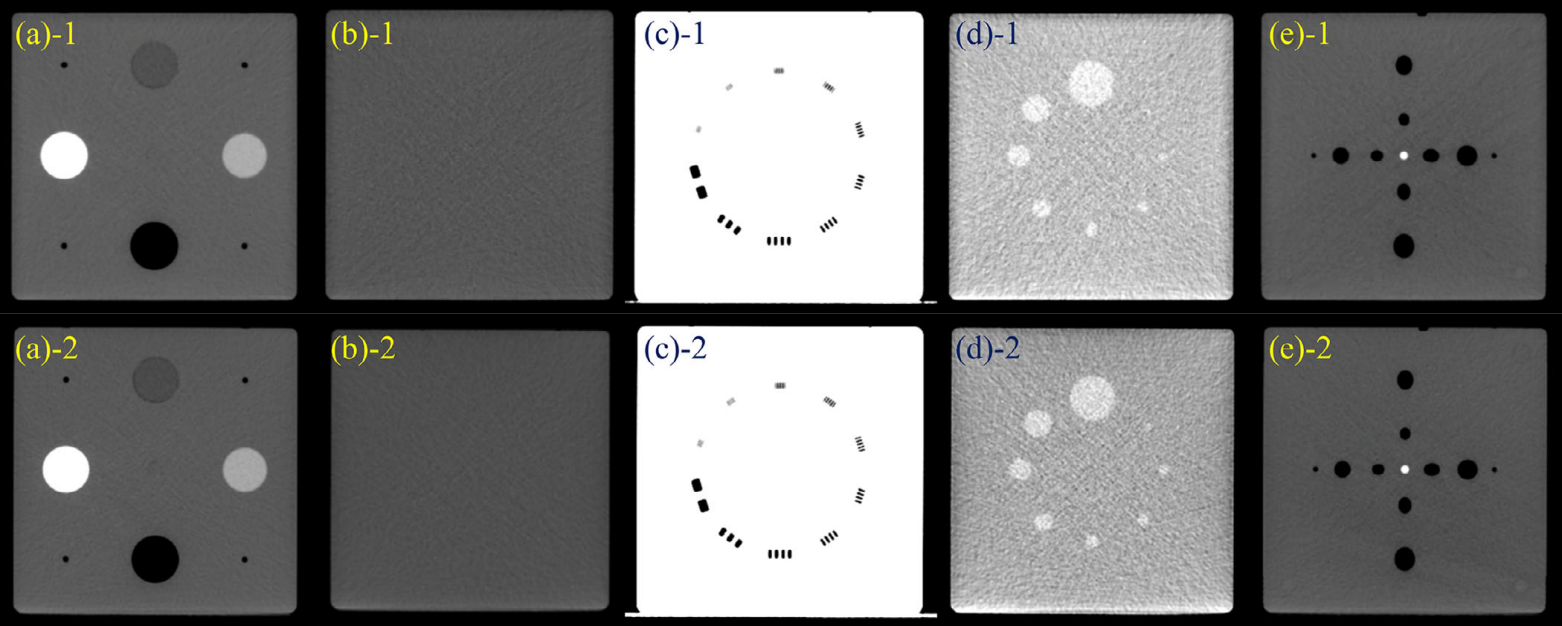
Transverse CT images from (1) Big Bore RT and (2) Discovery RT Gen3: (a) HU constancy, (b) uniformity, (c) spatial resolution, (d) low contrast, (e) coincidence check.

**FIGURE 4:**
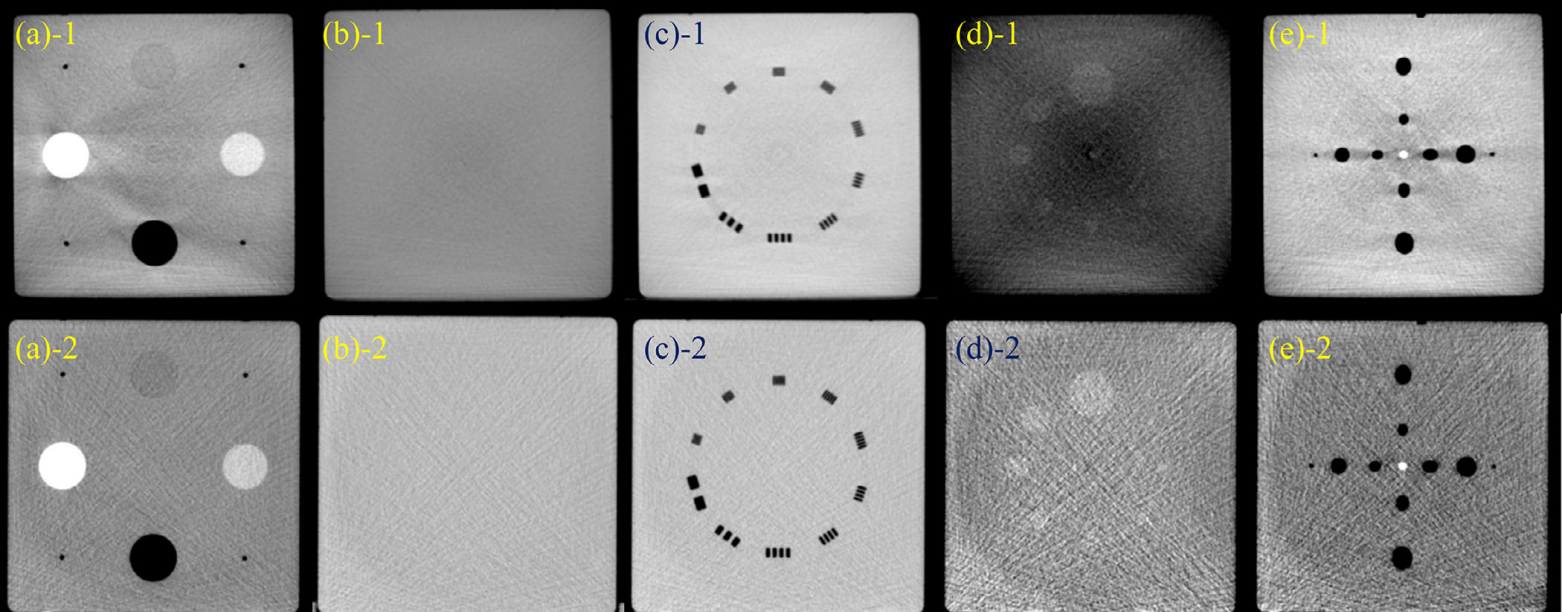
Transverse CBCT images from (1) XVI and (2) OBI: (a) HU constancy, (b) uniformity, (c) spatial resolution, (d) low contrast, (e) coincidence check.

Figures 3 and 4 show the MZ CUBE modules acquired from the two CT simulators and two CBCT systems, with evaluation results compared to commercial phantoms summarized in Tables 3. For HU constancy, differences between repeated measurements were within 3 HU across all systems. HU uniformity showed less than 1 HU variation in CT and up to 2 HU in CBCT. Spatial resolution and low‐contrast resolution matched the baseline values across all systems.

**TABLE 3:** Image quality assessment using MZ CUBE and commercial phantoms on CT and CBCT systems.

CT Systems	Phantom	Results	HU constancy (HU)					HU uniformity	Spatial resolution	Low contrast resolution (mm)
			Air	PE	Acrylic	Bone	Teflon	Max deviation	MLP^a^	mDD^b^
Big Bore RT	MZ CUBE	Baseline	−989.7	−51.2	128.2	N/A	963.9	3.0	7	7
		Measurement	−987.4	−52.2	127.8	N/A	966.3	2.7	7	7
	ACR 464	Baseline	−982.6	−83.7	127.5	895.5	N/A	1.9	7	25
		Measurement	−986.2	−84.0	128.0	895.7	N/A	3.3	7	25
Discovery RT Gen3	MZ CUBE	Baseline	−983.3	−63.3	116.3	N/A	959.2	1.1	8	5
		Measurement	−982.2	−64.6	116.5	N/A	960.3	1.8	8	5
	CatPhan 504	Baseline	−938.9	−81	122.3	N/A	896.5	2.1	10	5
		Measurement	−939.7	−82.1	123.9	N/A	897.1	1.7	10	5
XVI	MZ CUBE	Baseline	−877.2	−95.4	37.3	N/A	683.3	13.0	7	6
		Measurement	−874.3	−96.4	36.5	N/A	686.2	13.6	7	6
	ACR 464	Baseline	−892.2	−128.0	45.2	863.3	N/A	12.6	7	N/M^c^
		Measurement	−896.4	−126.2	44.2	864.6	N/A	11.4	7	N/M
OBI	MZ CUBE	Baseline	−980.2	−62.2	117.7	N/A	958.3	9.1	8	6
		Measurement	−981.9	−64.3	117.9	N/A	956.2	7.7	8	6
	CatPhan 504	Baseline	−998.3	−101.1	131.8	N/A	863.3	8.8	9	25
		Measurement	−996.5	−102.6	131.2	N/A	860.2	3.2	9	25

^a^ MLP: Maximum visible line pairs

^b^ mDD: Minimum discernible disk

^c^ N/M: Not measurable.

### 3.2 Geometrical accuracy assessment

Spatial integrity ranged from 9.99 to 10.02 cm. Imaging and treatment coordinate coincidence was within 0.2 mm, and positioning/repositioning errors are summarized in Table 4.

**TABLE 4:** Geometrical accuracy result with MZ CUBE and MIMI phantom.

Phantom	CBCT Systems		Offset					
			X (cm)	Y (cm)	Z (cm)	Pitch (°)	Roll (°)	Yaw (°)
MZ CUBE		Specification	2.70	2.40	2.00	1.30	0.80	1.00
	XVI	Measurement	2.74	2.37	1.95	1.40	0.60	1.20
	OBI	Measurement	2.69	2.30	2.04	1.20	0.80	0.90
MIMI Phantom		Specification	1.20	1.00	1.40	N/A		
	XVI	Measurement	1.12	1.10	1.50			
	OBI	Measurement	1.19	1.00	1.40			

### 3.3 Cross validation with other commercial phantoms

Tables 3 and 4 summarize image quality and geometrical accuracy results from MZ CUBE and commercial phantoms. HU differences between phantoms were generally within 10 HU, except for polyethylene and Teflon, where differences reached up to 30–60 HU. HU uniformity differences remained within 2 HU across all systems. Spatial resolution was comparable, except on Discovery RT Gen3, where CatPhan 504 achieved slightly higher resolution (10 lp/cm vs. 8 lp/cm). In low‐contrast resolution, MZ CUBE consistently detected smaller disks than the commercial phantoms, particularly on Big Bore RT and OBI CBCT.

### 3.4 Verification of miscellaneous details

**FIGURE 5:**
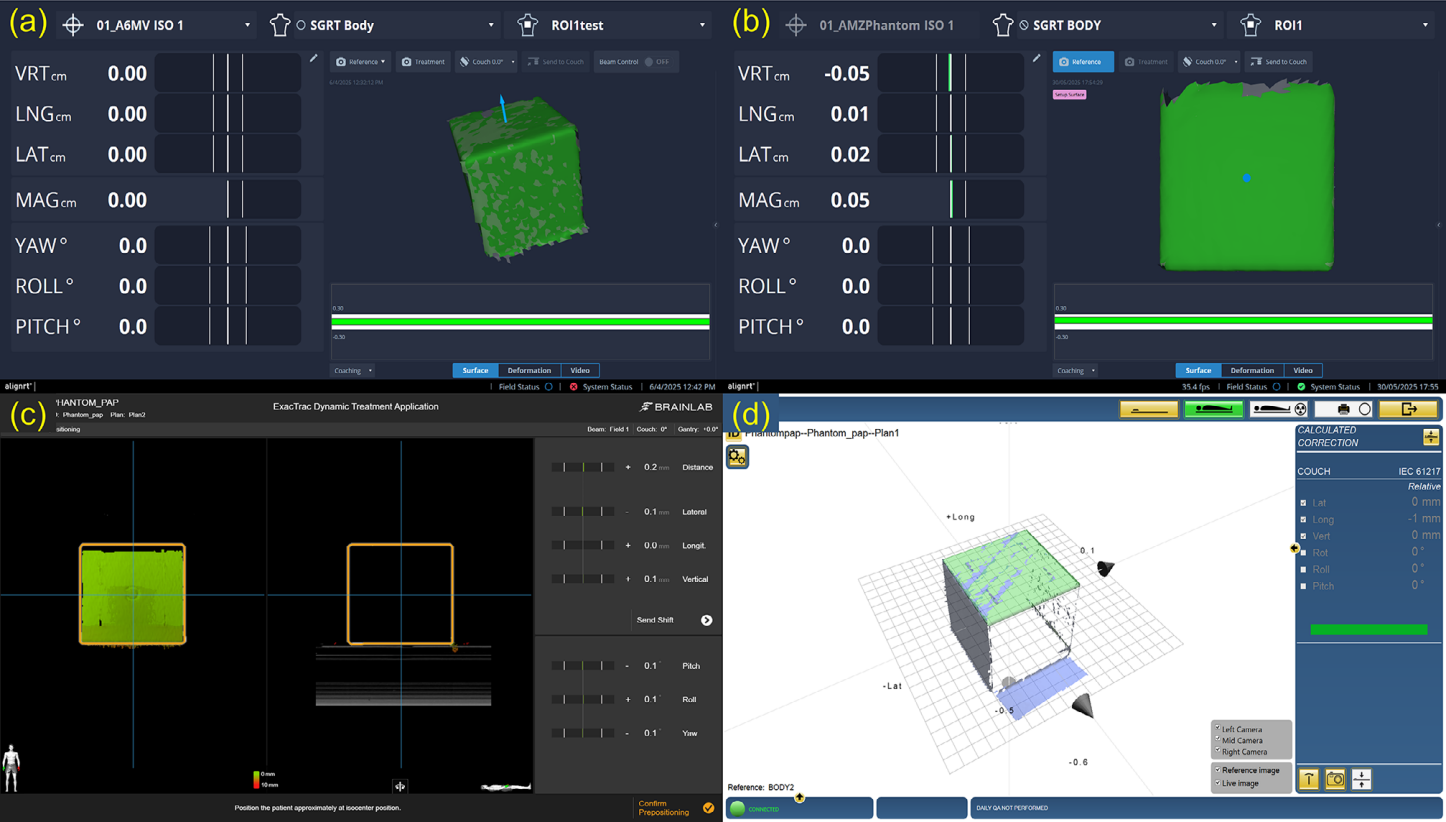
SGRT system interfaces showing phantom recognition: (a) AlignRT Gen5, (b) AlignRT Horizon, (c) ExacTrac Dynamic, (d) Catalyst^+^HD.

A 10 × 10 cm field at 100 cm SSD was visually confirmed for both linacs. All SGRT systems successfully recognized and tracked the MZ CUBE phantom. System interface screenshots demonstrating successful surface recognition by each SGRT platform are shown in Figure 5.

## 4 DISCUSSION

In this study, we developed the MZ CUBE phantom to enable integrated evaluation of both image quality and geometrical accuracy in IGRT, in accordance with the EFOMP‐ESTRO‐IAEA protocol.^5^ Unlike conventional approaches requiring separate phantoms and multiple scans, MZ CUBE combines both functions into a single device, reducing scan time and setup effort. It is applicable to both CT and CBCT systems, and tilt plates allow assessment of 6D couch geometrical accuracy. While setup time may vary by user, the consolidated workflow using a single phantom can significantly reduce overall QA time.

In the HU constancy module, the MZ CUBE employs air, polyethylene, acrylic, and Teflon to represent a wider range of electron densities. HU constancy differences were generally acceptable, though polyethylene and Teflon showed discrepancies of up to 30 HU and 60 HU, respectively, in CBCT compared to the CatPhan 504. These may result from differences in material composition and crystallinity, as well as CBCT‐specific artifacts such as scatter and beam hardening.^13^

For the low‐contrast module, previous studies used materials like PVC and Pinkysil silicone to slightly enhance contrast for low‐contrast evaluation.^14^, ^15^ In this study, VytaFlex was used for its similar contrast and moldability, enabling holes of various sizes. While the ACR 464 phantom has multiple low‐contrast holes, the large gap between 25 and 6 mm limits intermediate sensitivity. In contrast, the MZ CUBE includes 25–3 mm holes in finer steps, allowing detection of 6–7 mm disks and more precise evaluation across CBCT systems.

Spatial resolution can be assessed qualitatively by counting visible line pairs or quantitatively via modulation transfer function (MTF) analysis using high‐density materials.^10^ The MZ CUBE is intended for qualitative evaluation using 10 air‐filled line patterns.^14^ Unlike commercial phantoms that enhance contrast with high‐Z inserts, its low‐signal design reflects a task‐based approach, where task transfer functions may be more relevant than MTF.^10^, ^16^

Geometrical accuracy methods vary by phantom design; some use full‐length rods or hollow spheres for alignment.^17^ The MZ CUBE includes eight circular and elliptical holes arranged horizontally and vertically. Ellipses improve plane distinguishability over circular openings alone, enhancing alignment accuracy. Moreover, although the MZ CUBE was primarily developed for volumetric CT/CBCT QA, its centrally located circle is also visible on planar images, allowing for 2D–2D registration to verify setup accuracy.

This study has limitations. First, the MZ CUBE's cubic shape may cause non‐uniform attenuation and increased scatter near corners, unlike circular phantoms that ensure uniform path lengths during rotation. While this may affect consistency in acceptance testing, it has little impact on periodic QA focused on constancy. Second, the phantom does not support quantitative spatial resolution metrics like MTF, but remains suitable for routine QA using qualitative line patterns per AAPM TG‐142.

The MZ CUBE enables integrated CT and CBCT QA by assessing image quality and geometrical accuracy in a single scan, reducing reliance on separate phantoms. Its compatibility with four SGRT systems also supports its use in SGRT accuracy checks. Despite integrating multiple QA functions typically covered by separate commercial phantoms, the MZ CUBE remains cost‐competitive. It reduces QA‐related costs and streamlines logistics, offering a practical solution for diverse clinical settings.

## 5 CONCLUSIONS

We developed an integrated QA phantom compliant with the EFOMP‐ESTRO‐IAEA protocol that evaluates both image quality and geometrical accuracy in a single scan. Unlike conventional methods using separate phantoms, the MZ CUBE enables simultaneous assessment, providing time and cost efficiencies for radiotherapy QA.

## AUTHOR CONTRIBUTION

Minsoo Chun contributed to the conceptualization, methodology, investigation, validation, data interpretation, and writing of the original draft and review & editing. Jaewon Hong was responsible for data curation and validation. Jin Hwa Choi contributed to data curation and formal analysis. Chang‐Heon Choi was involved in the conceptualization, methodology, investigation, resources, data interpretation, and supervision. Jung‐in Kim contributed to the conceptualization, methodology, investigation, resources, data interpretation, supervision, funding acquisition, and project administration. Hong‐Gyun Wu worked on data curation, resources, formal analysis, supervision, and funding acquisition. Minjae Choi contributed to data curation, formal analysis, validation, and visualization. Na Young Jang assisted with data curation and formal analysis. So‐Yeon Park contributed to the conceptualization, methodology, investigation, validation, supervision, project administration, and writing of the review & editing. All the authors reviewed the results and approved the final version of the manuscript.

## CONFLICT OF INTEREST STATEMENT

The authors declare the following financial interests/personal relationships which may be considered as potential competing interests. Chang‐Heon Choi, Jung‐in Kim, Hong‐Gyun Wu, and Minjae Choi have financial relationships with Paprica Lab Co., Ltd. to disclose. The remaining authors declare no competing interests.
